# From Outbreak to Near Disappearance: How Did Non-pharmaceutical Interventions Against COVID-19 Affect the Transmission of Influenza Virus?

**DOI:** 10.3389/fpubh.2022.863522

**Published:** 2022-03-29

**Authors:** Shuxuan Song, Qian Li, Li Shen, Minghao Sun, Zurong Yang, Nuoya Wang, Jifeng Liu, Kun Liu, Zhongjun Shao

**Affiliations:** ^1^Department of Epidemiology, Ministry of Education Key Lab of Hazard Assessment and Control in Special Operational Environment, School of Public Health, Air Force Medical University, Xi'an, China; ^2^Department of Infectious Disease Control and Prevention, Xi'an Center for Disease Prevention and Control, Xi'an, China; ^3^School of Remote Sensing and Information Engineering, Wuhan University, Wuhan, China

**Keywords:** influenza, COVID-19, respiratory diseases, non-pharmaceutical interventions, long-term impact

## Abstract

Influenza shares the same putative transmission pathway with coronavirus disease 2019 (COVID-19), and causes tremendous morbidity and mortality annually globally. Since the transmission of COVID-19 in China, a series of non-pharmaceutical interventions (NPIs) against to the disease have been implemented to contain its transmission. Based on the surveillance data of influenza, Search Engine Index, and meteorological factors from 2011 to 2021 in Xi'an, and the different level of emergence responses for COVID-19 from 2020 to 2021, Bayesian Structural Time Series model and interrupted time series analysis were applied to quantitatively assess the impact of NPIs in sequent phases with different intensities, and to estimate the reduction of influenza infections. From 2011 to 2021, a total of 197,528 confirmed cases of influenza were reported in Xi'an, and the incidence of influenza continuously increased from 2011 to 2019, especially, in 2019–2020, when the incidence was up to 975.90 per 100,000 persons; however, it showed a sharp reduction of 97.68% in 2020–2021, and of 87.22% in 2021, comparing with 2019–2020. The highest impact on reduction of influenza was observed in the phase of strict implementation of NPIs with an inclusion probability of 0.54. The weekly influenza incidence was reduced by 95.45%, and an approximate reduction of 210,100 (95% CI: 125,100–329,500) influenza infections was found during the post-COVID-19 period. The reduction exhibited significant variations in the geographical, population, and temporal distribution. Our findings demonstrated that NPIs against COVID-19 had a long-term impact on the reduction of influenza transmission.

## Introduction

Influenza virus is an RNA-enveloped virus belonging to the Orthomyxoviridae family and causes a highly contagious acute respiratory illness. Approximately 3–5 million cases of severe illness and 290,000–650,000 deaths worldwide have been attributed to seasonal influenza annually (1). Many factors influence the geographic heterogeneity and seasonality of influenza, including temperature, relative humidity, and population flow (2–4), which are also responsible for the significantly different seasonality of influenza in northern and southern China, especially in crowded metropolitan cities (5). Vaccination is considered to be the most effective preventive measure for reducing the incidence and severity of influenza (6); however, it is still difficult to establish a strong community immunity against influenza, because of the high variability and the consequent uncertainty of the epidemic strain and the low vaccination rate (7, 8). Therefore, developing effective measures to prevent the transmission of influenza is essential.

Xi'an, located on the Silk Road in northwestern China, experienced a serious outbreak of influenza during 2018–2019. A multiple fold increase was observed in the number of influenza cases from 2011 to 2019, with a total number of 186,610 cases. During the influenza outbreak in 2019, there were 142,208 cases of influenza with an incidence rate of 109.79 per 10,000 population, which is nearly 100 times higher than that in previous years and 20 times higher than the national average incidence. It was predicted that influenza cases in Xi'an might reach an unprecedented peak in 2020 and even cause an epidemic. Xi'an is an internationally famous tourist city with a huge flow of people. Therefore, if an epidemic breaks out here, it would spread at a very fast speed and lead to serious consequences. However, a sudden outbreak of coronavirus disease 2019 (COVID-19) disrupted the transmission of influenza when the local government started taking strict measures to contain the spread of COVID-19. As Xi'an is adjacent to Hubei province, the focus of COVID-19 in the early stage of the pandemic, the city was seriously affected by COVID-19. To control and prevent the spread of COVID-19, various non-pharmaceutical interventions (NPIs) such as quarantine, disinfection of public places, closure of schools and offices, social distancing, closure of borders, travel curbs, and restrictions on going out were adopted. As a corollary to these measures, the number of influenza cases in 2020 also dramatically decreased, and a predictable outbreak seemed to disappear in Xi'an. It is reasonable to ratiocinate that NPIs were equally effective in containing the spread of influenza. Similar results have also been reported by many studies globally (9–13). The incidence of laboratory-confirmed influenza cases dropped by 65.02% in China (14). In Singapore, the influenza test positivity decreased by 64% due to COVID-19 (15). However, it is not clear (a) how did the influenza cases decrease so rapidly that the number of active cases became far lower than that in the pre-COVID-19 period; (b) how did NPIs against COVID-19 affect the epidemic of influenza; and (c) how would it affect the dynamic pattern of influenza in Xi'an both in the short and long terms. Very few studies have demonstrated the long-term influence of NPIs against COVID-19 on influenza; most of the studies have focused on the short-term impact only (9, 16). As the SARS-CoV-2 Delta Variant and Omicron Variant spread globally (17), it is crucial to quantify the NPIs on infectious diseases.

Here, we conducted the present retrospective epidemiological study using data on influenza in Xi'an from 2010 to 2021 (a) to identify and compare the seasonal pattern and epidemiological features of influenza between 2020 and 2021 influenza outbreak and that of previous years, (b) to lay a foundation for further investigation into the social factors that influence the influenza transmission, and (c) to assess the results of the measures adopted to fight against COVID-19 on the reduction in morbidity and mortality caused by the influenza virus.

## Materials and Methods

### Data Collection and Management

#### Influenza

We obtained weekly reports on the confirmed cases of influenza surveillance data from March 28, 2011 to December 31, 2021 from Xi'an Municipal Center for Disease Control and Prevention (CDC). All sentinel hospitals in the Chinese National Influenza Surveillance Network are required to report information on influenza cases to the Chinese National Influenza Surveillance Information System (CNISIS), as well as collect respiratory samples (throat swab and nasal swab) within 3 days of onset for influenza virus detection by using PCR. Demographic data were also obtained, including sex, date of birth, occupation, living address, and date of onset. The confirmed cases of influenza were confirmed by laboratory testing. We defined the 14th week to the 13th week of the following year as the influenza year to conduct annual statistics and analyze the influenza surveillance data (http://www.chinaivdc.cn/cnic/zyzx/jcfa/201709/t20170930_153976.html), and the 2021 influenza year was defined from the 14th week to the end of the year due to the lack of data of 2022. Based on the influenza test positivity rates, we categorized the average positivity across all epidemic weeks of a monitoring year into high (positive rate ≥ 25%), moderate (20–25%), and low (<20%) levels (9).

#### NPIs Against COVID-19

The emergence response against COVID-19 in China is divided into four levels based on the character, degree of harm, and impact scope. The Level I emergence response is the highest and would initiate the strictest public health interventions such as region lockdown, traffic restriction, social distancing, wearing masks, and compulsory health quarantine (http://www.nhc.gov.cn/). Since the first case of COVID-19 in Xi'an was reported on January 23, 2020, the local government of Xi'an implemented Level I emergence response on January 25, 2020 in the entire city (http://xawjw.xa.gov.cn/). As a result, the number of new confirmed cases and asymptomatic carriers of COVID-19 in Xi'an dropped to zero by February 22, 2020. Consequently, the emergence response was revised down to Level III on February 28, 2020 (http://xawjw.xa.gov.cn/), and schools, from kindergartens to colleges, resumed classes in Xi'an from June 8, 2020. People returned to their normal life similar to that in the pre-COVID-19 period, except for still wearing masks, doing nucleic acid testing and scanning QR codes. The reported cases were all imported from overseas until January 28, 2021 when a local case of transmission was confirmed, which prompted Xi'an government to immediately take intervention measures to stop community spread. Hence, we divided our study period into four phases: Pre-COVID19 Phase (from March 28, 2011 to January 24, 2020); Phase I (from January 25, 2020 to June 8, 2020); Phase II (from June 9, 2020 to January 17, 2021); and Phase III (from January 18, 2021 to December 31, 2021).

Web search index, such as Google Flu Trends, has been successfully applied for improving the prediction of influenza (18, 19). Baidu Search Index (BSI) from the shared platform of Baidu (https://index.baidu.com/), the biggest Chinese search engine, has become the most used trend analysis data in China. The BSI of specific keyword indicates the normalized search volume, which can provide dynamic trend about the search behaviors for disease symptom and treatment of online users in different regions. To measure public attention to influenza, we selected five related keywords with detailed description in Supplementary Table 1 and obtained BSI value of these keywords within Xi'an from March 28, 2011 to December 31, 2021. Weather data in Xi'an, including temperature, relative humidity, precipitation, evaporation, atmospheric pressure, and sunshine duration, were obtained from the Meteorological Data Sharing Service System. Vector boundaries of Xi'an were obtained from the basic geographic database in the National Catalog Service for Geographic Information of China (https://www.webmap.cn).

### Data Analysis

#### Descriptive Analysis

To compare differences in the dynamic pattern of influenza cases between pre- and post-COVID-19 phases, descriptive statistical methods were used to analyze the annual influenza cases and influenza epidemic strain from 2011 to 2021. Sex, age group (0–3 years; 3–6 years; 6–15 years; 15–18 years; 18–60 years; ≥60 years) and counties of cases helped to gain detailed results as classifications.

#### Statistical Analysis

To assess how influenza was affected by NPIs, we utilized a composite approach for time-series analysis, including quantifying the causal impact of NPIs, predicting the counterfactual number of influenza cases, and investigating the quantitative effects of interventions emerging in the post-COVID-19 phase. We constructed a Bayesian structural time-series (BSTS) model, which is widely used to predict infectious diseases, to try and predict the counterfactual number of influenza cases after the outbreak of COVID-19 (20–22). The BSTS model produced better forecasts with the following advantages: it allows the inclusion of prior information and the model parameters to evolve over time; it is less dependent on certain hypothesized specifications; and it assumes that the relationship between covariates and time series remains stable throughout the post-period of an infection (23). Markov chain Monte Carlo (MCMC) algorithm helps to simulate posterior probability distribution and Gibbs sampling algorithm was used to generate posterior samples from conditional distribution for predicting. We ran five chains with 20,000 iterations and discarded 4,000 initial iterations as burn-in each (80,000 effective samples in total) for BSTS model. By integrating the outpatient data derived from the pre-COVID-19 phase with the predicting model, we were able to predict the epidemic dynamics with two types of covariates as regression components in the post-COVID-19 phase when no preventive measures were taken. A more detailed model using meteorological factors was built to illustrate the epidemic pattern in different counties and age groups. To estimate the prediction accuracy, we calculated the symmetric mean absolute percentage error (SMAPE) and root mean square error (RMSE) of different models.

We used interrupted time series analysis (ITSA) to determine the effectiveness of interventions. ITSA is a powerful analysis method to investigate time series data during trend shifting caused by an interruption or intervention. It uses the segmented regression approach to analyze the time series data during two phases: before and after the intervention (24). Given that the seasonal pattern has been controlled in the seasonality component of BSTS, we applied ITSA as the regression component with adjusting for autocorrelation. To assess the impact of NPIs in different stages, we set three time points of intervention changes corresponding with Phase I to Phase III. Three indicator variables representing intervention changes were coded 0 in the pre-intervention period and 1 in the post-intervention period, respectively. The regression coefficients of indicator variables represent level changes of influenza incidence, and slope changes following interventions were also measured by combining with continuous time variable. The posterior distribution and inclusion probabilities of regression coefficients were used to estimate the impact of NPIs on influenza and select the best predictors.

All analyses were conducted with the R version 4.0.4. The BSTS model was constructed by using the “bsts” version 0.9.7 and “CausalImpact” packages version 1.2.7 was used to analyze the causal impact of the influenza test positivity rate. The thematic maps of average annual influenza cases during 2011–2017 and 2018–2019 and relative reduction in 2020 in Xi'an were produced by using ArcGIS version 10.8 software.

### Ethical Statement

The influenza surveillance is a governmental public health task in the charge of the Xi'an Municipal CDC. Therefore, an ethical review by an ethics committee was not required.

## Results

### Characteristics of Influenza Epidemics

**Figure 1:**
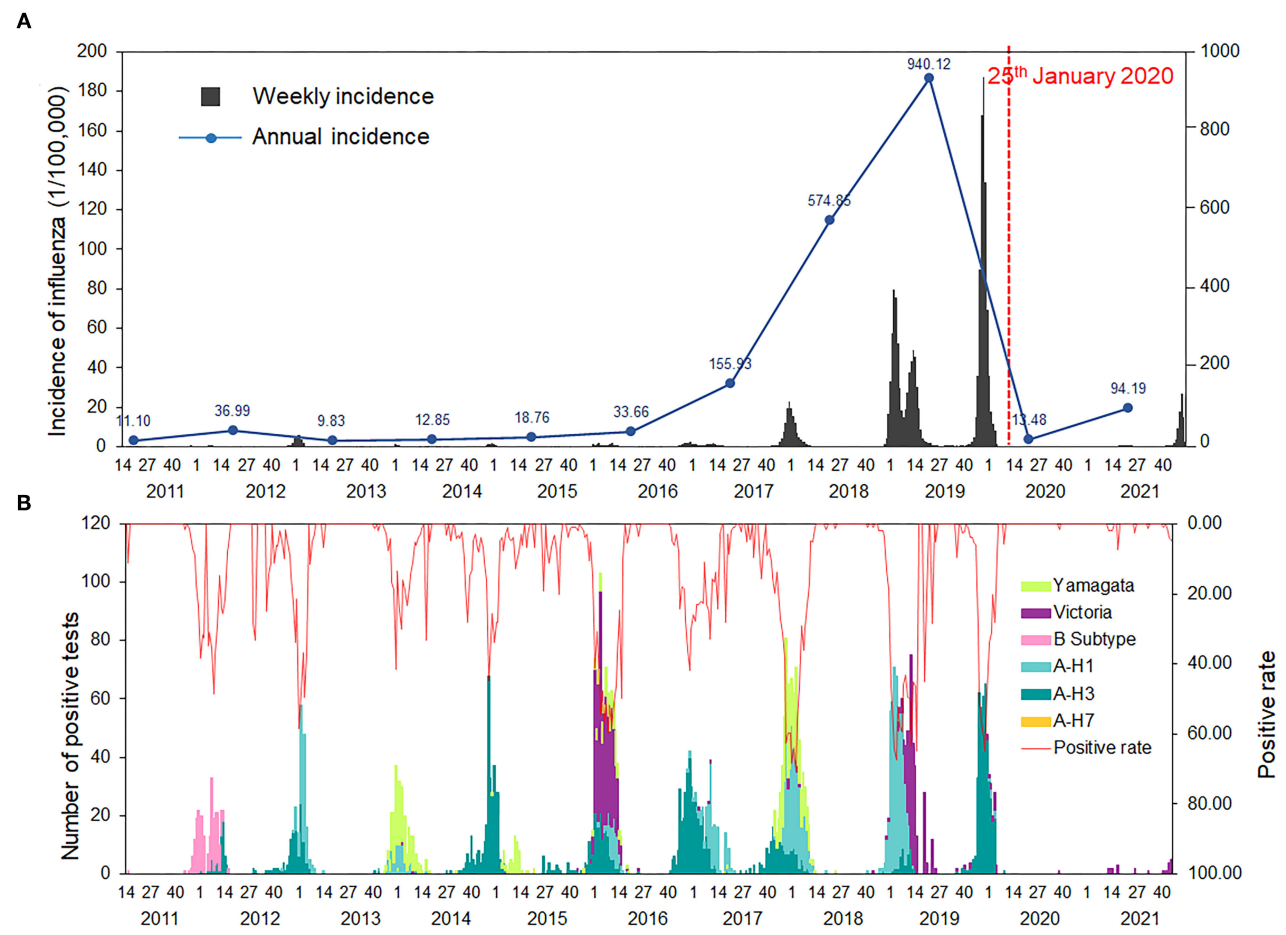
Temporal distributions of influenza in Xi'an, 2011–2021. **(A)** Weekly and annual incidence of influenza in Xi'an, from 2011 to 2021. **(B)** Number of positive tests and positive rate of influenza reported by laboratories in Xi'an, from 2011 to 2021.

From March 28, 2011 to December 31, 2021, 197,528 influenza cases were confirmed and 30,908 pharyngeal swabs were collected in Xi'an. Among these samples, 4,987 tested positively for the influenza virus by PCR. Figure 1 illustrates that influenza cases in Xi'an increased every year from 2013 to 2019, especially in 2018–2019 and 2019–2020, with 56,029 and 99,539 cases, respectively. However, after the implementation of NPIs on January 25, 2020, only 2,308 cases of influenza were reported in 2020–2021—a decrease in the incidence of 97.68%. Even the influenza incidence reduced by 87.22% in 2021, comparing with that of 2019–2020. An annual periodicity in winter/spring was noted during the entire surveillance period, except for 2018–2019, which had a year-round periodicity. Additionally, we observed different predominant pathogens, among which the influenza A virus was the most predominant one (Figure 1).

**Figure 2:**
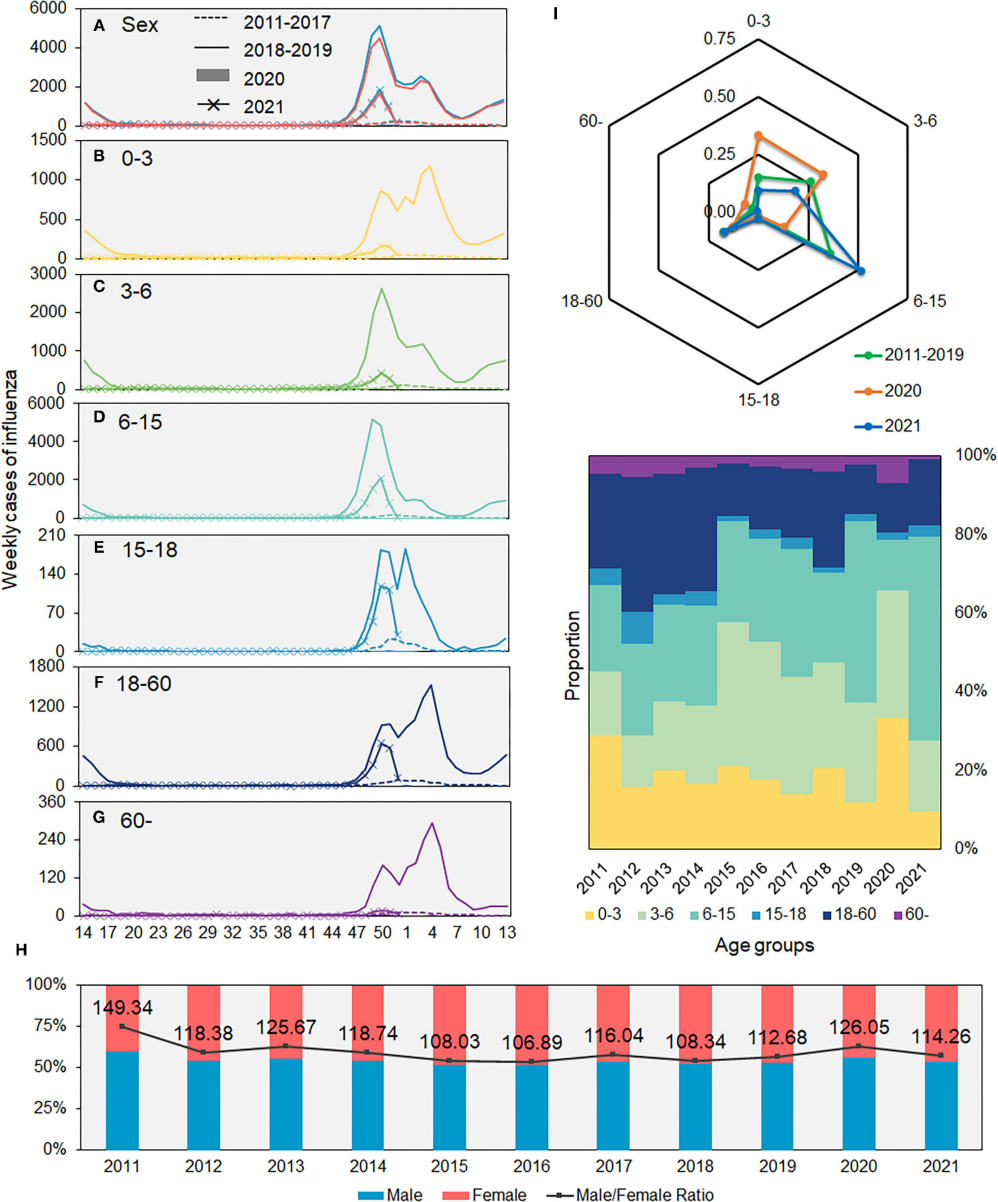
Average weekly number of influenza cases in Xi'an, 2011–2021. **(A)** Sex. **(B–G)** Different age groups. **(H)** Proportions of male and female patients. **(I)** Proportions of influenza cases in different age groups.

We divided the study period before the COVID-19 pandemic into two: 2011–2017 and 2018–2019. The proportion of the affected children aged 6–15 years (*n* = 72,407, 36.66%) was highest among the six age groups. However, the proportion of patients of all age groups changed in 2020–2021; younger children of age 0–3 years and 3–6 years accounted for 34.01% and 28.77% of the total patients during 2020–2021, respectively (Table 1). The epidemic dynamic pattern of all age and sex groups was different between 2011–2017 and 2018–2019. Overall, the epidemic peak during 2018–2019 happened earlier than that in previous years, and the patients belonging to the 0–3 years group shared the same pattern with that of the 18–60 years and older than 60 years group, with two peaks, while the 3–6 and 6–15 years groups had a similar pattern (Figure 2). The epidemic pattern of 15–18 years group combined the younger and adult groups. The onset time was similar with 3–6 and 6–15 years groups but with two epidemic peaks.

**Table 1:** Demographic characteristics of the confirmed cases of influenza from Chinese National Influenza Surveillance Information System (CNISIS) in Xi'an from 2011 to 2021.

**Characteristics**	**2011–2012**	**2012–2013**	**2013–2014**	**2014–2015**	**2015–2016**	**2016–2017**	**2017–2018**	**2018–2019**	**2019–2020**	**2020–2021**	**2021**
No. patients	1,018	3,310	901	1,152	1,656	3,158	15,691	56,045	99,576	2,308	12,713
Incidence rate (1/100,000)	12.02	38.88	10.53	13.36	19.02	35.76	163.16	560.24	975.90	17.82	98.15
**Sex**, ***n*** **(%)**	
Male	614 (60.31)	1,803 (54.47)	513 (56.94)	637 (55.30)	856 (51.69)	1,653 (52.34)	8,438 (53.77)	29,142 (52.00)	52,762 (52.99)	1,293 (56.02)	6,771 (53.26)
Female	404 (39.69)	1,507 (45.53)	388 (43.06)	515 (44.70)	800 (48.31)	1,505 (47.66)	7,253 (46.23)	26,903 (48.00)	46,814 (47.01)	1,015 (43.98)	5,942 (46.74)
**Age group (years)**, ***n*** **(%)**			
0–3	316 (31.04)	560 (16.92)	178 (19.76)	192 (16.67)	348 (21.01)	581 (18.40)	2,212 (14.10)	11,744 (20.95)	11,843 (11.89)	785 (34.01)	1,238 (9.74)
3–6	166 (16.31)	434 (13.11)	152 (16.87)	233 (20.23)	593 (35.81)	1,114 (35.28)	4,634 (29.53)	14,937 (26.65)	25,356 (25.47)	664 (28.77)	2,395 (18.84)
6–15	215 (21.12)	766 (23.14)	227 (24.08)	283 (24.57)	437 (26.39)	803 (25.43)	4,938 (31.47)	12,692 (22.65)	45,295 (45.45)	285 (12.35)	6,504 (51.16)
15–18	40 (3.93)	259 (7.82)	22 (2.44)	43 (3.73)	23 (1.39)	68 (2.15)	448 (2.86)	864 (1.54)	3,318 (3.33)	43 (1.86)	368 (2.89)
18–60	233 (22.89)	1,114 (33.66)	283 (31.41)	362 (31.42)	220 (13.29)	506 (16.02)	2,882 (18.37)	13,484 (24.06)	11,592 (11.64)	352 (15.25)	2,103 (16.54)
60+	45 (4.42)	174 (5.26)	49 (5.44)	39 (3.39)	35 (2.11)	86 (2.72)	577 (3.68)	2,324 (4.15)	2,197 (2.21)	179 (7.76)	105 (0.83)

**Figure 3:**
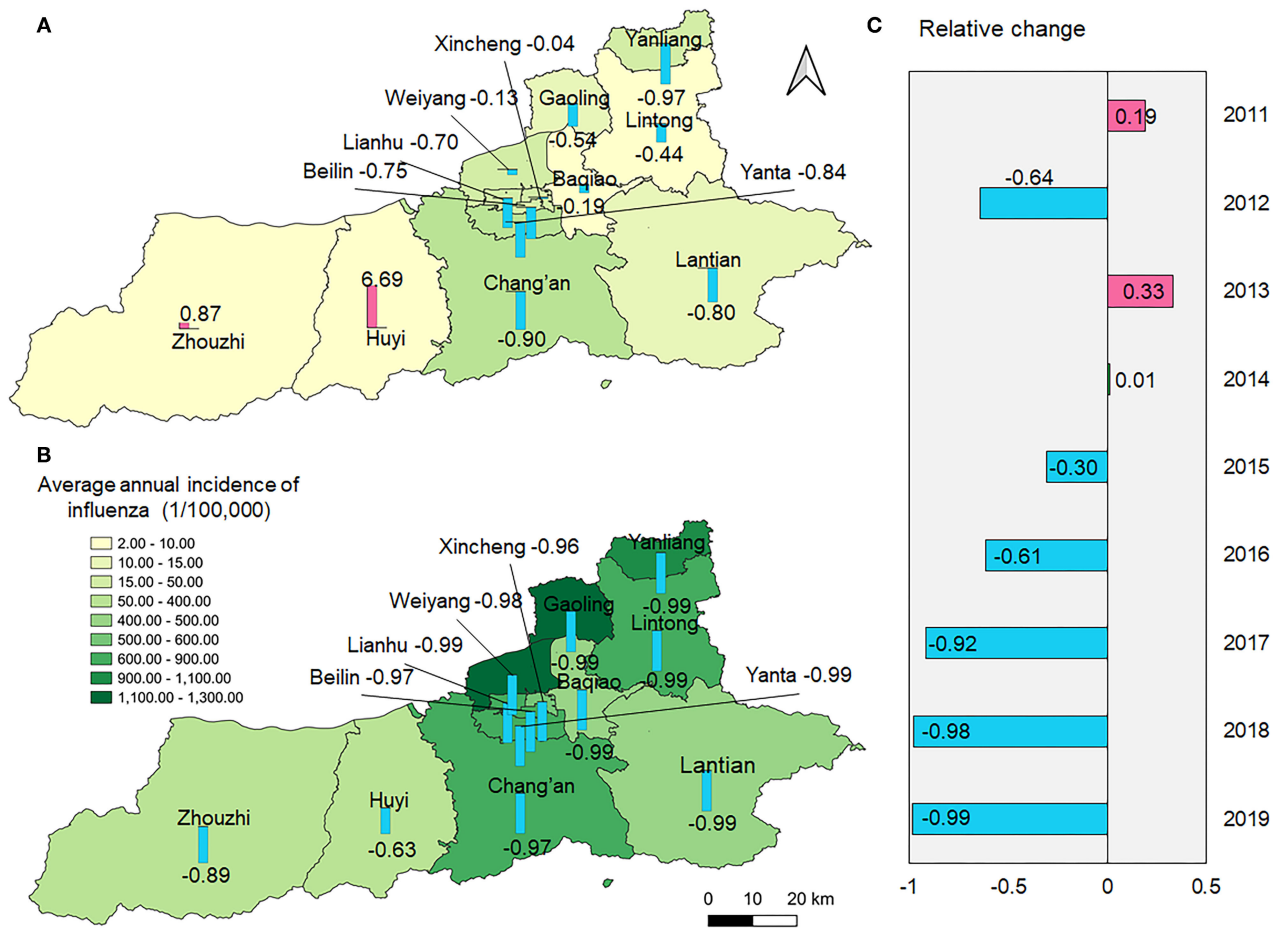
Spatial distribution of average annual incidence of influenza during 2011–2017 and 2018–2019 and relative reduction in 2020–2021. **(A)** The spatial distribution of average annual incidence of influenza during 2011–2017 and the relative reduction comparing with 2020; **(B)** The spatial distribution of average annual incidence of influenza during 2018–2019 and the relative reduction comparing with 2020–2021; **(C)** Relative change of the incidence of influenza between 2020–2021 and the previous 9 years, respectively.

All counties of Xi'an had a similar epidemic pattern, except for Zhouzhi and Huyi counties, in which the influenza incidence rates were higher in 2020–2021 than that in 2011–2017 (Figure 3; Supplementary Table 2). In addition, the influenza cases in 2020–2021 were higher than the average number in week 14–40 and lower than that in week 41–13 when compared with the average number from 2011 to 2017 and were below that of 2018–2019.

### Impact of NPIs Against COVID-19 on Influenza

**Figure 4:**
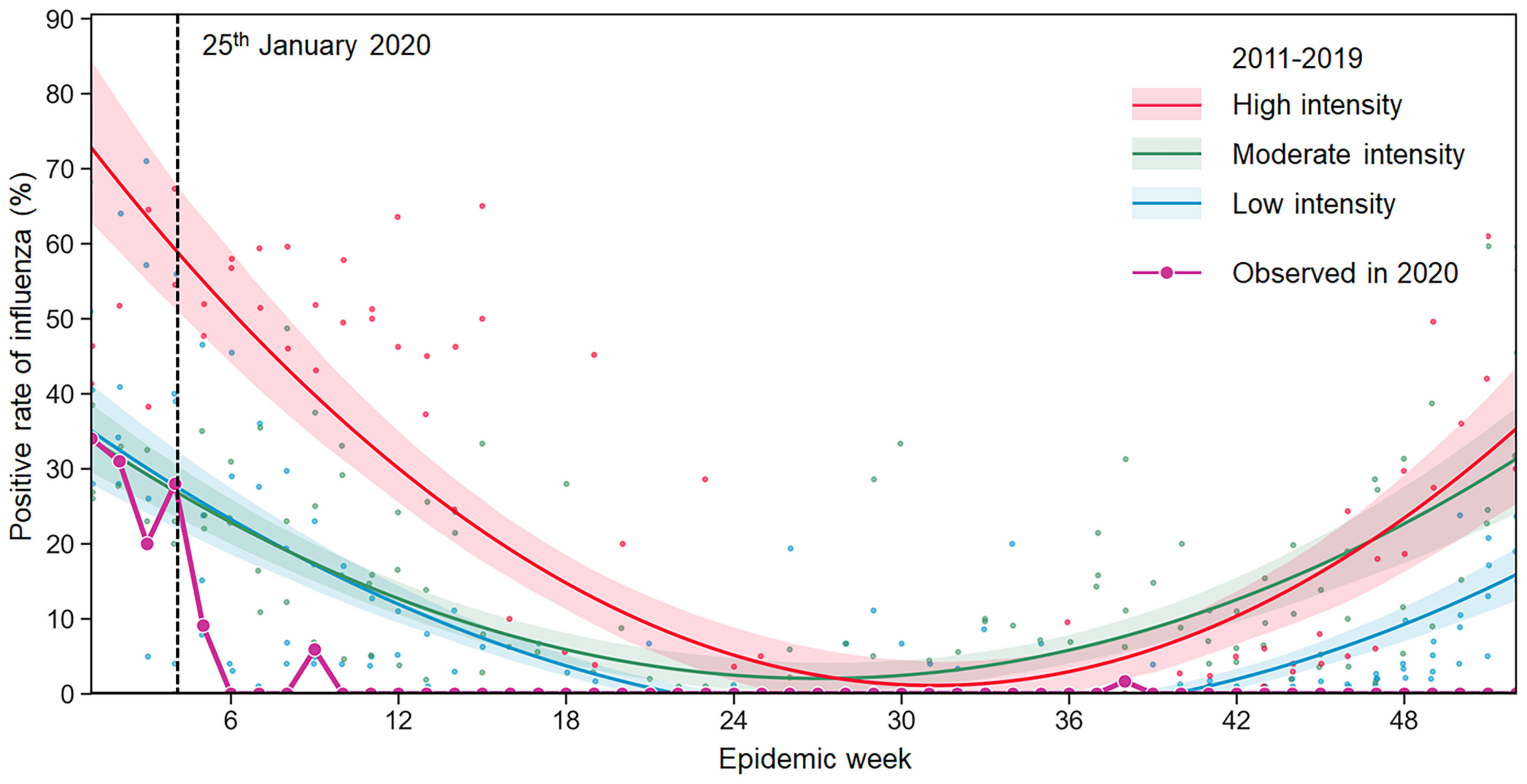
Observed seasonal influenza epidemic in 2020 and predicted levels using the influenza surveillance data of 2011–2019. The intensity of influenza activity was divided into three levels in China: high, moderate, and low, corresponding to high (≥25%), moderate (20–25%), and low (<20%) average test positivity rates for all epidemic weeks within a monitoring year from 2011 to 2019. The fitted curve for each intensity level is presented with lower and upper bounds (shaded color). The black-dotted line indicates when the NPIs were implemented in Xi'an.

**Figure 5:**
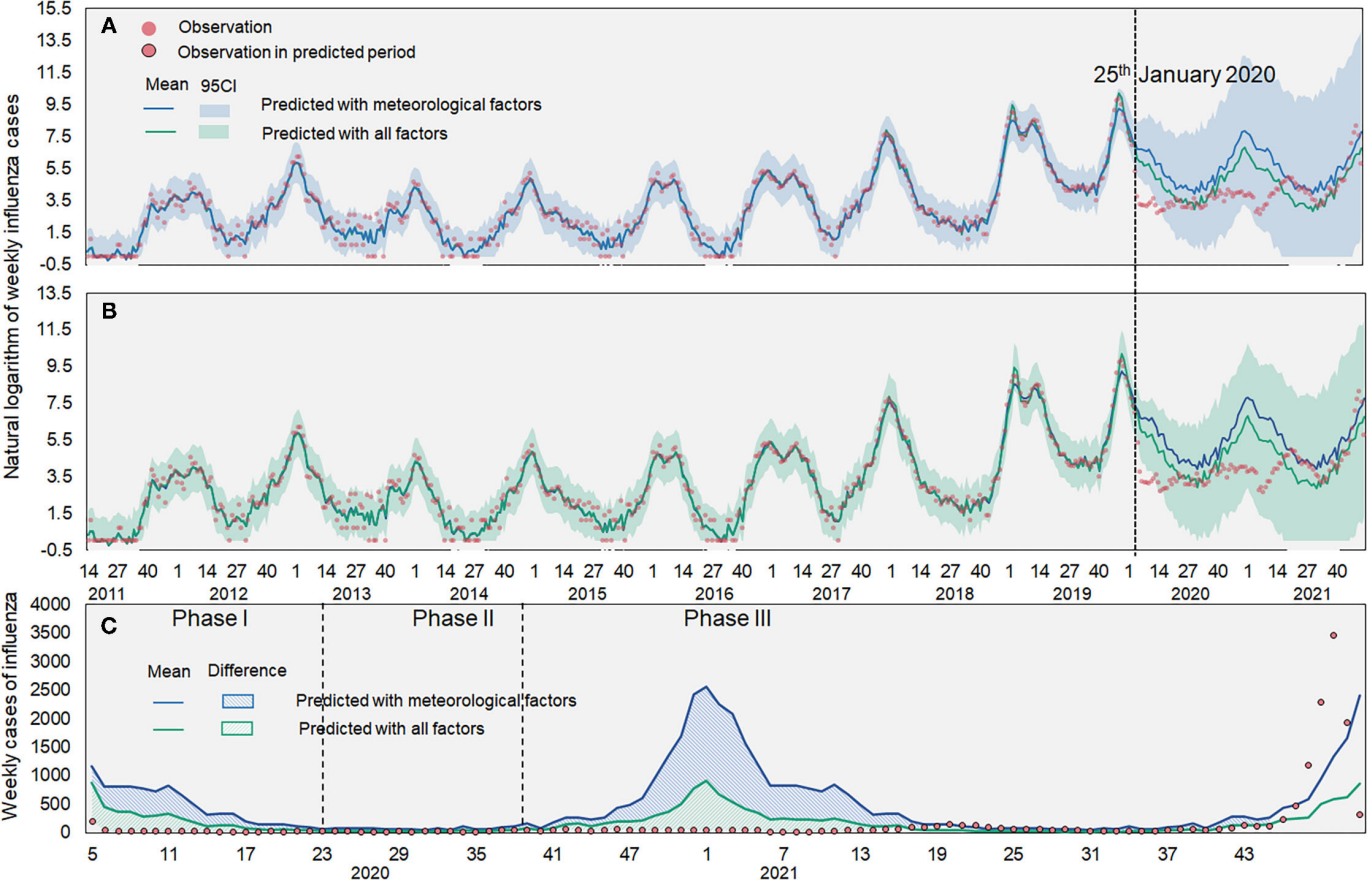
Observed and predicted influenza cases in Xi'an. **(A)** Predicted influenza cases based on meteorological factors; **(B)** Predicted influenza cases based on meteorological factors and BSI; **(C)** Difference between observed and predicted cases. The red dot denotes the observed influenza cases. The blue line denotes the predicted influenza cases with meteorological factors and the green line denotes the predicted influenza cases with BSI and meteorological factors. The shaded denotes 95% confidence intervals of the predicted value.

From January 25, 2020, Xi'an initiated the highest-level public health emergence response against COVID-19. As a result, the influenza test-positive rate dramatically decreased from moderate to low (Figure 4). To assess the impact of NPIs, we built BSTS models to fit influenza activities from 2011 to 2019 and predict the influenza epidemic levels in 2020–2021. One-step ahead prediction accuracy indicated that these models could be used for subsequent analyses (SMAPE: 0.058; RMSE: 0.371). As the number of influenza cases in 2018–2019 far exceeded that in previous years, a logarithmic transformation was performed on the number of influenza cases from 2011 to 2021 for a more accurate and reliable prediction. The prediction made using BSI and meteorological factors was more accurate than that made using meteorological factors only. The results demonstrated that the observed influenza cases were significantly lower than the predicted ones, especially in Phase II when another epidemic peak of influenza was expected (Figure 5). The model was used to predict the trends of influenza in each county during 2020–2021. The results showed that the counties showed different seasonal trends, although all the trends had an epidemic peak in the winter of 2020–2021. The cumulative differences compared with the actual values are summarized in Table 2 and Supplementary Figure 1.

**Table 2:** Cumulative difference between the observed and predicted influenza cases in three post-COVID-19 phases in Xi'an, 2020–2021.

	**Time period**					
	**Phase I**		**Phase II**		**Phase III**	
	**Absolute difference**	**Relative difference**	**Absolute difference**	**Relative difference**	**Absolute difference**	**Relative difference**
Xi'an	−8897.66 (−112912.14, −245.59)	−0.90 (−0.99, −0.20)	−20783.96 (−2291983.98, 841.42)	−0.95 (−1.00, 3.78)	−3624.57 (−6551305.13, 12307.20)	−0.23 (−1.00, 247.15)
Xincheng	−571.50 (−7075.62, −34.20)	−0.91 (−0.99, −0.38)	−867.11 (−52643.27, 48.07)	−0.93 (−1.00, 2.68)	−577.69 (−167631.56, 332.47)	−0.63 (−1.00, 50.89)
Beilin	−917.63 (−11443.34, −81.79)	−0.96 (−1.00, −0.66)	−1346.57 (−100916.34, 14.64)	−0.97 (−1.00, 0.72)	−1077.60 (−330431.28, 278.52)	−0.79 (−1.00, 37.21)
Lianhu	−1269.24 (−11573.42, −159.5)	−0.97 (−1.00, −0.80)	−1885.54 (−84816.24, 14.38)	−0.97 (−1.00, 0.31)	−910.90 (−225999.34, 882.42)	−0.50 (−1.00, 50.19)
Baqiao	−539.99 (−7370.56, −49.66)	−0.95 (−1.99 −0.66)	−660.62 (−43830.87, 15.67)	−0.96 (−1.00, 1.27)	−478.23 (−178342.11, 333.99)	−0.59 (−1.00, 66.72)
Weiyang	−2021.98 (−19149.33, −196.95)	−0.95 (−0.99, −0.63)	−3450.08 (−141187.80, 55.45)	−0.96 (−1.00, 0.59)	−2137.14 (−377331.91, 1203.45)	−0.63 (−1.00, 33.85)
Yanta	−1116.49 (−18415.99, −54.07)	−0.92 (−0.99, −0.37)	−2299.14 (−361558.96, 73.83)	−0.96 (−1.00, 4.30)	−428.14 (−1181869.11, 1320.34)	−0.24 (−1.00, 360.62)
Yanliang	−481.81 (−6459.38, −49.66)	−0.96 (−1.00, −0.72)	−537.63 (−34086.09, −10.07)	−1.00 (−1.00, −1.00)	−631.36 (−155505.01, 69.59)	−0.90 (−1.00, 15.77)
Lintong	−1492.99 (−22316.64, −139.86)	−0.97 (−1.00, −0.76)	−1595.54 (−115654.58, −9.09)	−0.99 (−1.00, −0.36)	−2048.05 (−564800.08, 92.20)	−0.95 (−1.00, 8.53)
Chang'an	−996.76 (−15658.46, −41.28)	−0.93 (−1.00, −0.35)	−1943.95 (−242303.02, 59.79)	−0.96 (−1.00, 3.28)	978.85 (−811081.80, 2631.36)	0.59 (−1.00, 567.64)
Lantian	−739.18 (−14337.54, −45.71)	−0.95 (−1.00, −0.57)	−972.02 (−112610.72, 4.07)	−0.99 (−1.00, 0.41)	−851.91 (−470488.31, 207.77)	−0.80 (−1.00, 64.26)
Zhouzhi	−1141.69 (−5718.52, −213.11)	−0.95 (−0.99, −0.77)	−1382.40 (−13559.73, −87.58)	−0.95 (−0.99, −0.53)	−1354.70 (−29415.16, 383.88)	−0.73 (−0.98, 3.09)
Huyi	−329.12 (−1689.96, −65.25)	−0.91 (−0.98, −0.66)	−250.20 (−3565.40, 58.54)	−0.72 (−0.97, 1.56)	−285.83 (−10175.07, 185.29)	−0.57 (−0.98, 6.24)
Gaoling	−1050.28 (−15273.51, −105.15)	−0.98 (−1.00, −0.80)	−1057.49 (−80581.33, −4.77)	−0.99 (−1.00, −0.30)	−741.40 (−400877.34, 730.72)	−0.50 (−1.00, 100.34)

**Figure 6:**
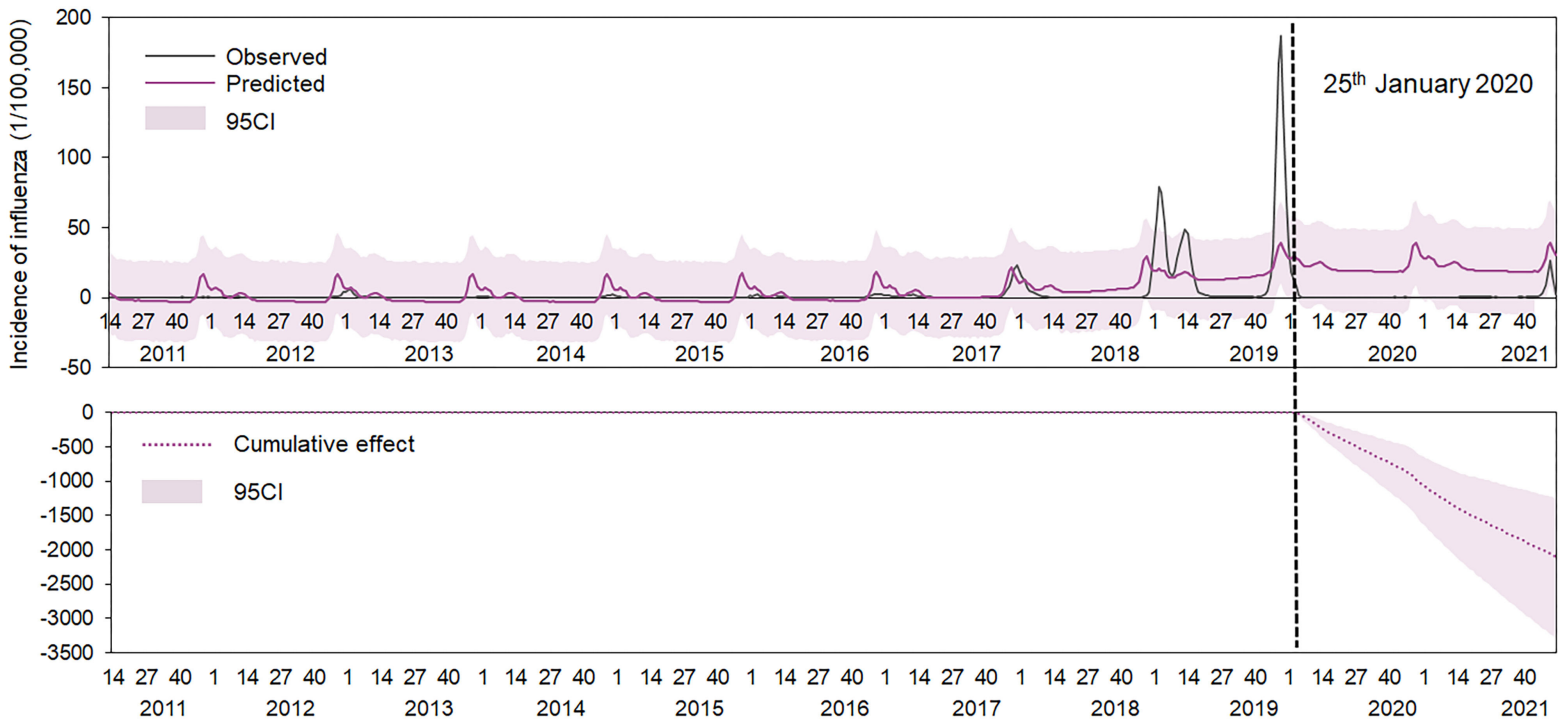
Observed and predicted incidence of influenza from 2011 to 2021, Xi'an. The gray line denotes the observed value. The red line denotes the predicted value and the black-dotted line denotes 25 January 2020, when the NPIs were implemented in China; the red-dotted line denotes the cumulative incidence between the observed and the predicted incidence, and pink shades denotes 95% confidence intervals of the predicted value.

The ITSA results revealed that the slope change TX1 in the long-term trend after Phase I had the highest impact on the reduction of influenza cases (inclusion probability: 0.54) and its β5 coefficient is negative, followed by level change X1 in the beginning of Phase I (inclusion probability: 0.52) with a negative coefficient (Table 3). To assess the long-term impact of NPIs on seasonal influenza, we projected an influenza dynamic pattern in 2020–2021 using influenza incidence data and compared the predicted incidence under the counterfactual scenario with the actual incidence. The results illustrated that the weekly influenza incidence in Xi'an declined by 95.45% and an approximate reduction of 210,100 influenza cases was found [95% confidence interval (CI): 125,100–329,500] during post-COVID-19 phase since the implementation of NPIs in Xi'an (Figure 6; Table 4).

**Table 3:** Statistics of the regression coefficient of variables in ITSA.

	**Coefficients**			**Standardized coefficients**	**Inclusion probability**
	**Mean**	**2.5%**	**97.5%**		
β1(T)	−0.00	0.00	0.00	0	0.02
β2(X1)	−1.62	−3.74	0.49	−0.26	0.52
β3(X2)	−3.04	−11.14	5.06	0	0.02
β4(X3)	0.66	−0.95	2.28	0.04	0.07
β5(TX1)	−0.00	−0.00	0.00	−0.20	0.54
β6(TX2)	0.01	−0.01	0.02	0.41	0.02
β7(TX3)	0.00	−0.00	0.00	0.05	0.07

**Table 4:** Potential impact of COVID-19 and non-pharmaceutical interventions on seasonal activity in Xi'an.

	**Actual value**	**Prediction**	**Absolute effect**
Average	1.1	22 (14, 34)	−21 (−33, −13)
Cumulative	111	2,212 (1,362, 3,406)	−2,101 (−3,295, −1,251)

## Discussion

Influenza is an acute respiratory disease that causes tremendous morbidity and mortality annually worldwide. Our study showed that an outbreak of influenza in 2020 in Xi'an was curbed by NPIs adopted to contain the transmission of COVID-19.

We noted a dramatic increase in the incidence of influenza over the past decade with an outbreak during 2018–2019 in Xi'an, which was in line with other studies that showed that the number of influenza cases in other areas continued to increase in the past decade (5, 25). Several factors could be responsible for this increase. Firstly, insufficient vaccination rate might lead to the increased incidence, as the influenza vaccination of China averaged 1.5–2.2% (26) and the decline of vaccination coverage was associated with the influenza cases rise (27). Secondly, enhanced influenza surveillance network and protocol might also result in increased incidence of influenza. Previous study has demonstrated that new update of the influenza surveillance protocol was related to a 65.6% increase in the influenza incidence risk in 2017 (5), and enhanced influenza surveillance network might report more cases of influenza to CNISIS. Thirdly, rapid variability of influenza viruses allows for the reinfection of previously infected or vaccinated individuals and leads to the lack of immunity to the current epidemic strain (7, 28). The result showed that the influenza incidence in 2011–2017 was much lower than that in 2018–2019, which was consistent with other studies by Zhu et al. (29) and Wu et al. (30). Since the influenza pandemic occurred in 2009, Xi'an has not experienced a similar large-scale epidemic in later years, and the diversified influenza epidemic spectrum showed co-circulated dynamics, resulting in a generally low level of population immunity. In addition, the influenza viruses in 2017–2018 were influenza B/Yamagata and influenza A/H1, which was different from that in 2016–2017 and 2018–2019, leading that the incidence of influenza in 2017–2018 and 2018–2019 increased significantly compared with the previous year. The epidemic of influenza in Xi'an tends to peak in winter and the predominant viruses change over time, which can be partly explained by host susceptibility, limited immunological cross-reactivity between influenza subtypes, and climatic factors (31, 32). A previous study showed that influenza A H3N2 variant viruses (H3N2v), responsible for influenza cases of 2016–2017, had a higher antigenic drift and drug resistance than those of international vaccine strains (33), which might be partly responsible for the increased prevalence of H3N2v during 2019–2020. Predominant influenza strains in Xi'an showed an alternate co-circulated pattern. Influenza A was the predominant strain during 2018–2020, and influenza B should be the predominant in 2020–2021 according to the previous epidemic pattern. However, we observed that both influenza A and B showed significant decline, and only a few cases of influenza B were detected in the laboratory, suggesting that NPIs might have a more significant effect on influenza A. This result was consistent with previous study (34). We found that the incidence of influenza in 2021, from the 14th week to the last week of 2021, was higher than that of 2020–2021. The NPIs implemented in the first half of 2020 were very strict, including home isolation, social distancing and delaying the start of school, etc. At the beginning of the resumption of work, due to the severe epidemic situation as before, people would subconsciously abide by the previous NPIs. However, such self-consciousness would change over time, making it usual to not wear masks when going out. Furthermore, the decline in the level of immunity to influenza in the population, and the different influenza strains in 2021 from the previous year might also contribute to the increasing incidence in 2021.

Our results demonstrated that men are more likely to be affected by influenza than women, consistent with the results of previous studies (35–37). Societal and behavioral factors, gender differences in immune response to vaccines, and compliance with prevention and control measures can influence exposure and susceptibility to viruses differently in men and women (38–40). The 0–3 and 3–6 years groups had a higher susceptibility to influenza viruses than that of any other age groups. Most of the children under 3 years have not entered schools, and their societal relationships were simple, as they are in contact with their families only, while children aged 3–18 years spend most of their time in schools, with a more complex social network and in more crowded spaces. Previous studies have proven that the influenza virus transmits rapidly with a higher incidence of human contact, and schools are important social environments that can facilitate rapid transmission of influenza via touching contaminated surfaces and close contact among students (41–43). Although both the 6–15 and 15–18 years groups spent most of their time in school, the influenza cases of 6–15 years group were much higher than that of the 15–18 years group, which might due to the immature immune system of children. These factors might explain why the 6–15 years group accounted for the highest number of cases and the 0–3 years group had the same epidemic pattern with that of the adult group.

To control the rapid transmission of COVID-19, a series of aggressive and extensive NPIs were implemented by the local government. Many studies have reported that the influenza incidence decreased during COVID-19 (9–13). Our study showed that a potential outbreak of influenza in 2020 was curbed by the outbreak of COVID-19. The observed influenza cases in 2020–2021 decreased dramatically compared with those in the past decades as well as compared to the expected numbers in 2020–2021. This result is similar to that reported by other studies (9–13, 43, 44). Qi et al. quantified the influence and reported a reduction of more than 60% in the incidence of both influenza A/H1 and B (34). In Guangdong, Xiao et al. found that influenza decreased by 95.1% compared with the expected numbers (44). The magnitude of reduction in Xi'an was different when compared with the number of cases in 2011–2017 and 2018–2019; it was still higher than that of mainland China, probably because of the strict and aggressive NPIs as Shaanxi province adjoins Hubei province, the epicenter of COVID-19 in the early stage. Our results also revealed that the largest reduction in the number of influenza cases was observed in the 6–15 year age group. Typically, children in this age group contributed to the largest proportion of influenza cases in Xi'an. However, as part of NPIs against COVID-19, schools were not opened until June 2020, which reduced the opportunity of coming in close contact with others. Previous studies have also highlighted the importance of school closure for controlling the transmission of infectious diseases (45, 46). A study in the U.S. demonstrated that school closure is associated with 62% reduction in the incidence and 58% reduction in mortality caused by COVID-19 (47). Additionally, children and parents paid more attention to personal hygiene, which further decreased the transmission of influenza. We predicted the epidemic of influenza at the county level and noted spatial heterogeneity in the relative reduction of influenza. We noted that the influenza incidence rates of Zhouzhi county and Huyi county in 2020–2021 were higher than that in 2011–2017. As Xi'an experienced rapid urbanization, the population of Xi'an has increased by 52.97% in the past decades, especially in the central area of the city. Zhouzhi and Huyi have the lowest population density, so the impact of NPIs such as social distancing and avoiding gathering implemented in these counties might be paid less attention to than that in center urban areas. In addition, the confirmed cases of COVID-19 of Xi'an in phase I were mostly concentrated in the center areas such as Beilin and Xincheng counties, while Huyi and Zhouzhi reported only a few. This might lead to the different implementation of specific NPIs in these counties as well as the differences in people's intentions to seek medical care when they find themselves with clinical symptoms. Furthermore, distribution of accessible health services, population susceptibility and distribution of school can also contribute to the spatial heterogeneity.

In addition, the ITSA result demonstrated that the NPIs implemented in Phase I had the highest impact on influenza; we observed the largest reduction in the test-positive rate during Phase II, but not during Phase I, which might due to the behavior change as a result of the implementation of NPIs. This result was different form a study of Guangdong province that the largest reduction was observed during weeks 9–19 (44). People started avoiding gatherings, wearing masks, reducing travel, and paying more attention to personal hygiene. Although various social and economic activities restarted in Phase II, sporadic cases of COVID-19 were still reported every few months in China, and strict NPIs were implemented discontinuously, leading to persistent low levels of influenza, which demonstrated that intermittent NPIs may play an effective role in controlling the transmission of respiratory infectious diseases. It was predicted that an epidemic peak of influenza would occur in the winter of 2020 and 2021 when schools closed for the winter holiday and Spring Festival, which might explain why the relative reduction in influenza cases during Phase II was higher. However, most studies of influenza reduction during COVID-19 were concentrated on the overall decreased incidence, and few studies have reported the specific phase after the implementation of NPIs when the reduction of incidence of influenza was largest. Further studies are needed. We observed a large reduction in the influenza incidence during 2020–2021, possibly because people became more conscious toward their health and started taking measures to stop the community spread of the virus (48). This observation emphasizes the long-term impact of NPIs on the transmission of infectious diseases.

The present study has several limitations as well. Firstly, the data did not include whether the patients had taken vaccines. The lack of availability of vaccination data and antigenic analysis in our study population limited our possibility to determine the factual long-term effect of NPIs and draw credible inference on this topic. Secondly, concerns regarding the SARS-CoV-2 outbreak might have changed the detection of influenza through changes in symptomatic individuals seeking medical attention or in physicians' inclination to test for influenza, which might result in the underreporting of influenza. Thirdly, the impact of NPIs on influenza in our study was indirect because it is difficult to acquire specific data on NPIs such as local consumption of masks and disinfectants.

## Conclusion

Our study revealed that NPIs against COVID-19 have both significantly short-term and long-term impacts on the reduction of influenza cases, and the impacts have heterogeneity in population, time and space. In addition, the findings suggested that effective prevention and control measures targeting the 6–15 years age group can reduce the number of influenza cases. Discontinuous implementation of strict NPIs may also help in keeping the influenza epidemic at a low level.

## Data Availability Statement

The raw data supporting the conclusions of this article will be made available by the authors, without undue reservation.

## Author Contributions

ZS, KL, and SS: concept and design. KL, QL, SS, JL, MS, and NW: acquisition and analysis of data. SS and MS: statistical analysis. SS, KL, and LS: drafting of manuscript. ZS, KL, LS, SS, and MS: revision of the manuscript. All authors have approved the final version of the manuscript.

## Funding

This work was supported by the National Natural Science Foundation of China (Grant Numbers: 81803289 and 42071368), the Natural Science Foundation of Shaanxi Province (Grant Number: 2020JM-329), and the 2022 National Innovation Training Program for college students at Wuhan University (B.S.).

## Conflict of Interest

The authors declare that the research was conducted in the absence of any commercial or financial relationships that could be construed as a potential conflict of interest.

## Publisher's Note

All claims expressed in this article are solely those of the authors and do not necessarily represent those of their affiliated organizations, or those of the publisher, the editors and the reviewers. Any product that may be evaluated in this article, or claim that may be made by its manufacturer, is not guaranteed or endorsed by the publisher.

## Abbreviations

COVID-19: coronavirus disease 2019
NPIs: non-pharmaceutical interventions
CDC: Center for Disease Control and Prevention
CNISIS: Chinese National Influenza Surveillance Information System
BSI: Baidu Search Index
BSTS: Bayesian structural time-series
MCMC: Markov chain Monte Carlo
SMAPE: symmetric mean absolute percentage error
RMSE: root mean square error
ITSA: interrupted time series analysis.
