# Supplementary material for: From Outbreak to Near Disappearance: How Did Non-pharmaceutical Interventions Against COVID-19 Affect the Transmission of Influenza Virus?
Source: Front Public Health. 2022 Mar 29;10:863522. doi: 10.3389/fpubh.2022.863522 (PMC9001955; doi:10.3389/fpubh.2022.863522)
Supplement: Supplementary file 1 [file Data_Sheet_1.PDF]

Supplementary materials

Table S1. Meteorological factors and Search key words from Baidu Index website used in the study.

	Variable	Min	Mean	Median	Max	Std.Dev	Definition in English
**Meteorological Factors**	Average temperature (C °)	-4.09	15.45	16.74	32.03	9.68	
	Relative humidity (%)	26.86	60.82	60.79	93.57	13.01	
	Precipitation (mm)	0.00	1.58	0.50	18.44	2.59	
	Evaporation (mm)	0.63	3.55	3.35	8.54	1.72	
	Atmospheric pressure (hPa)	952.57	968.43	968.64	986.79	8.14	
	Sunshine duration (h)	0.00	5.38	5.36	11.81	2.58	
**BSI**	Index 1	8.00	138.03	130.00	1085.00	98.17	Influenza
	Index 2	0.00	78.76	61.00	749.00	79.08	Influenza vaccine
	Index 3	0.00	48.08	41.00	182.00	31.63	Influenza virus
	Index 4	0.00	71.87	56.00	813.00	90.12	Flu symptoms
	Index 5	0.00	53.83	49.00	411.00	33.10	Influenza

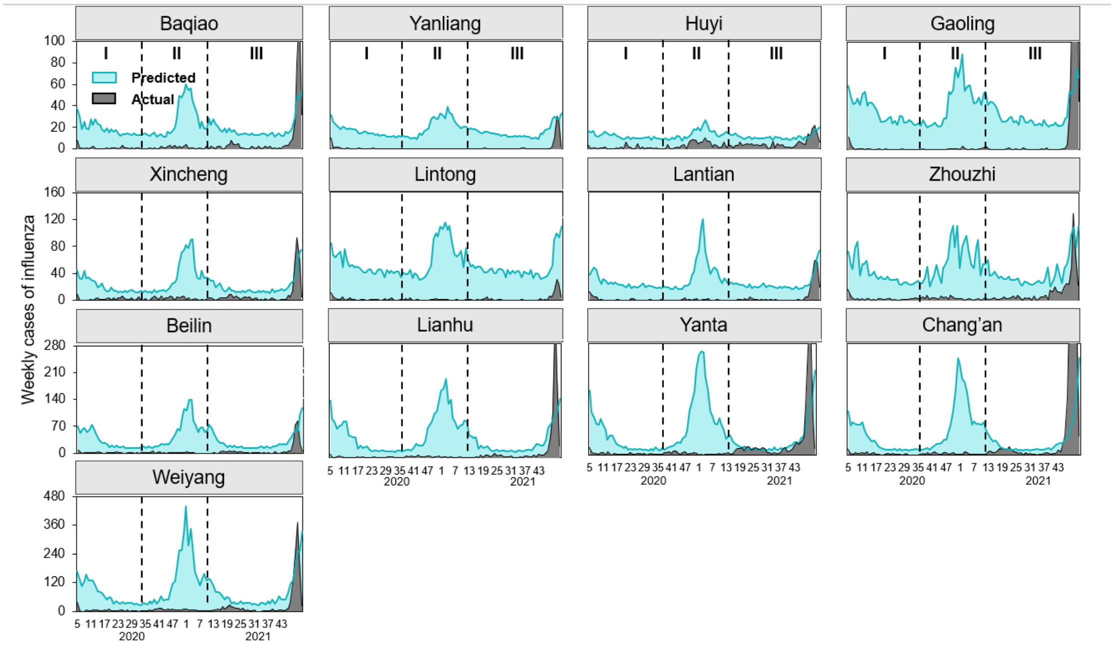

Figure S1. Observed and expected trends of seasonal influenza stratified by county in Xi'an in 2020–2021.
