# Supplementary material for: From Outbreak to Near Disappearance: How Did Non-pharmaceutical Interventions Against COVID-19 Affect the Transmission of Influenza Virus?
Source: Front Public Health. 2022 Mar 29;10:863522. doi: 10.3389/fpubh.2022.863522 (PMC9001955; doi:10.3389/fpubh.2022.863522)
Supplement: Supplementary file 2 [file Data_Sheet_2.PDF]

Table S2. Relative change in the number of influenza cases in different seasons between 2020 and 2021 and the average of 2018–2019 and 2011–2017. (14–40w: weeks 14–40; 41–30w: weeks 41 to week 13 of next year)

		2011-2017			2018-2019		
		Total	14-40w	41-13w	Total	14-40w	41-13w
Sex	Male	-0.51	2.78	-0.69	-0.98	-0.89	-0.98
	Female	-0.55	2.88	-0.73	-0.98	-0.90	-0.99
Age	[0,3)	-0.02	4.96	-0.39	-0.95	-0.83	-0.97
	[3,6)	-0.44	2.35	-0.62	-0.97	-0.89	-0.98
	[6,15)	-0.79	1.26	-0.86	-0.99	-0.96	-0.99
	[15,18)	-0.78	1.66	-0.86	-0.98	-0.84	-0.99
	[18,60)	-0.70	2.05	-0.84	-0.98	-0.92	-0.99
	[60,)	-0.11	5.80	-0.61	-0.95	-0.67	-0.98
Address	Xi’an	-0.67	1.40	-0.79	-0.98	-0.91	-0.99
	Xincheng	-0.04	6.69	-0.46	-0.96	-0.73	-0.98
	Beilin	-0.75	1.09	-0.88	-0.99	-0.90	-0.99
	Lianhu	-0.70	0.64	-0.81	-0.99	-0.95	-0.99
	Baqiao	-0.19	4.83	-0.43	-0.99	-0.89	-0.99
	Weiyang	-0.13	11.10	-0.45	-0.98	-0.88	-0.99
	Yanta	-0.84	0.74	-0.91	-0.99	-0.93	-0.99
	Yanliang	-0.97	6.00	-1.00	-1.00	-0.99	-1.00
	Lintong	-0.44	6.00	-0.76	-0.99	-0.87	-1.00
	Chang'an	-0.90	-0.44	-0.93	-0.99	-0.96	-0.99
	Lantian	-0.80	0.81	-0.87	-0.99	-0.86	-0.99
	Zhouzhi	0.88	5.60	0.22	-0.89	-0.32	-0.93
	Huyi	6.69	31.67	5.39	-0.63	-0.11	-0.68
	Gaoling	-0.54	7.75	-0.63	-0.99	-0.99	-1.00
